# Last but not yeast—The many forms of *Cryptococcus neoformans*

**DOI:** 10.1371/journal.ppat.1011048

**Published:** 2023-01-05

**Authors:** Piotr R. Stempinski, Gracen R. Gerbig, Seth D. Greengo, Arturo Casadevall

**Affiliations:** Department of Molecular Microbiology and Immunology, Johns Hopkins School of Public Health, Baltimore, Maryland, United States of America; University of Maryland, Baltimore, UNITED STATES

## Introduction

Most microscopic fungal species respond to their surrounding conditions with morphological changes, including transitions between yeast, pseudohyphal, and hyphal forms. In some cases, cells may undergo more subtle yet effective transformations that alter the size, shape, and biophysical barriers of the cells [1]. *Cryptococcus neoformans* is an encapsulated human fungal pathogen and the etiological agent of cryptococcosis and cryptococcal meningitis [2]. Cryptococcal infections begin with the inhalation of basidiospores, which initiate mostly asymptomatic lung infections in immunologically intact hosts [3]. In immunocompromised patients, infections can disseminate to the central nervous system (CNS), promoting life-threatening meningitis. In recent years, evidence has accumulated that morphological changes are key mechanisms that allow *C*. *neoformans* to persist in tissues in the form of enlarged cells and disseminate within the host organism as microcells [4].

### Mating and hypha formation

**Fig 1:**
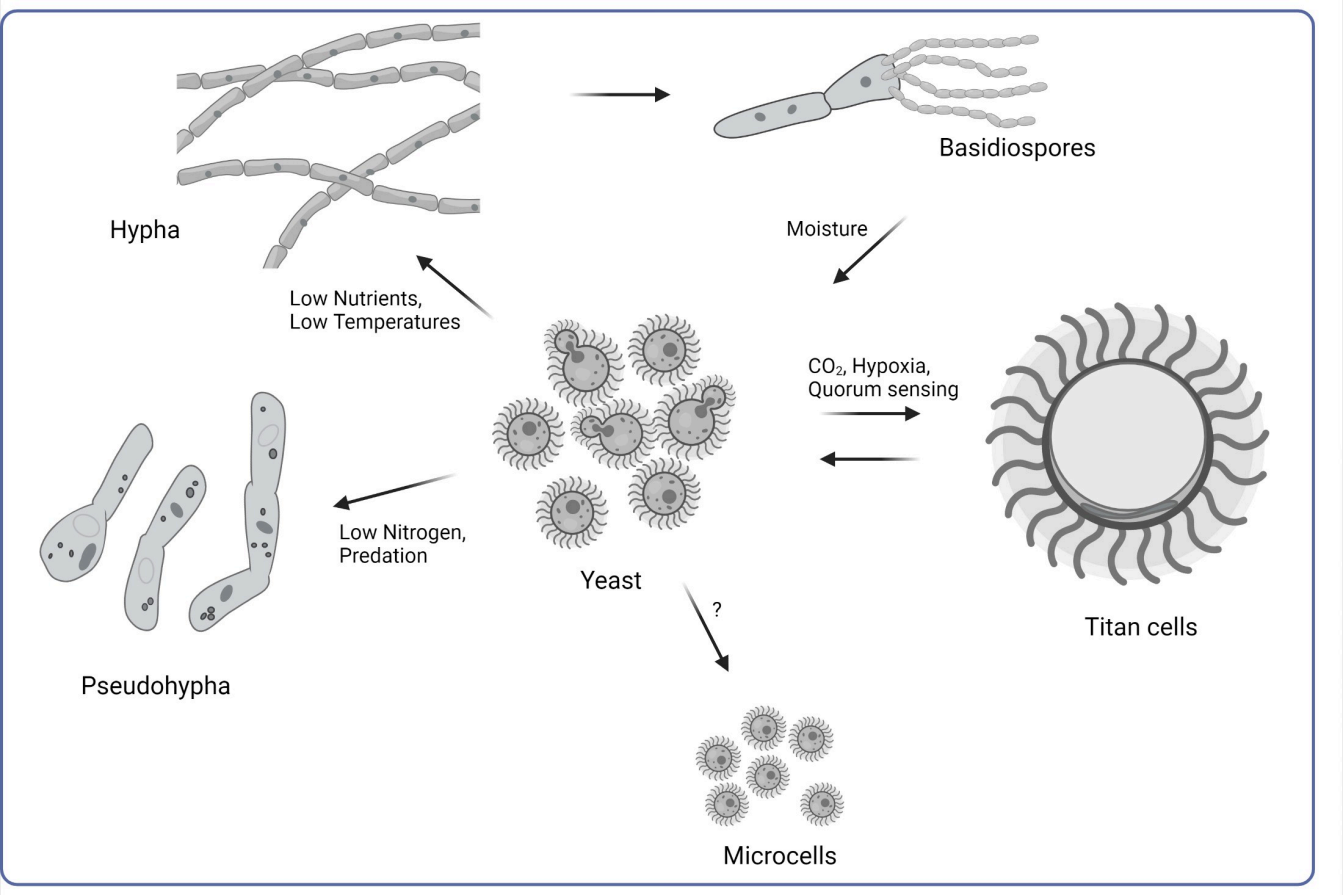
Illustration of the morphological changes of *Cryptococcus neoformans* cells. *C*. *neoformans* adapts to the surrounding environment by adjusting the cell morphology in response to various stimuli, therefore increasing the chance of survival in the natural environment or improving the virulence within the host.

The mating cycle of *C*. *neoformans* produces hyphae, a result of the fusion of two yeast cells. These branching segments later form basidia and spores (Fig 1). *C*. *neoformans* is a heterothallic fungus, with two mating types of yeast cells (MATa and MATα) [5]. The bipolar mating system is distinguished by two alleles at the MAT locus. Contact between MATa and MATα mating types produces zygotes that undergo filamentous growth. If opposite mating types, the hyphae are dikaryotic and parental nuclei remain separate; if same mating type, the hyphae are monokaryotic and the parental nuclei undergo fusion to produce diploids. [6]. Both types of fruiting can lead to basidiospore production. Transition to hyphal morphotypes is triggered by environmental changes, such as low temperature and moisture, as well as limited nutrient availability [6]. While pseudohyphae display resistance to amoeba and phagocytic predators, it is unknown whether the sexual hyphal forms of *C*. *neoformans* are resistant to phagocytosis. Interestingly, mating and the formation of hyphae are influenced by the nutrients sourced from the *Cryptococcus* environmental niche. For instance, *C*. *neoformans* can be sourced from bird guano and trees, and mating can be stimulated using a variety of media formulations, including plant-derived media, as well as live plants [7,8]. This suggests that the availability and source of nutrients drive sexual reproduction, which can increase genetic variety and lead to enhanced survival and virulence [7,9]. Recently, identified genes that are important to hyphal formation include *OLP1*, which produces an oxidoreductase-like protein and is necessary for proper hyphae formation, and *MKT1*, which is involved in pheromone gene expression and hyphal growth [10,11].

### Basidiospores

Basidia are specialized cells that are generated from the apical cell in the hyphae. Fusion of parental nuclei and subsequent meiosis and mitosis yield four chains of basidiospores (Fig 1) [12]. Aerosolized cryptococcal spores (approximately 3 μm) are infectious propagules that lodge within alveolar spaces in the lungs and germinate into yeast form. Several proteins regulate cryptococcal sporulation. In addition to deformed hyphal formation, *OLP1* deletion also blocks spore production [10]. The F-box protein *CDC4*, like the previously identified *FBP1*, is required to produce spores, possibly through regulation of meiosis [13,14]. Gluconate metabolism, as shown through deletion of the gene for the gluconate kinase *GNK1*, is also crucial in the spore production process [15]. Unlike spore production, regulation of the spore germination process is less studied. Huang and colleagues identified 18 proteins that were enriched in *C*. *neoformans* spores compared to yeasts and identified one, Isp2, as necessary for germination [16].

### Morphological transition to titan cells

One of the most unique and characteristic abilities of cryptococcal cells is the capacity for dramatic changes in size of the encapsulated cells (Fig 1). These enlarged “titan cells” are characterized by large cell bodies with a diameter ranging from 10 μm up to 100 μm, with a significantly thicker cell wall surrounded by a huge and tightly constructed capsule [17–19]. The intracellular space is almost entirely composed of a large vacuole with peripherally located nucleus and cell organelles [17,20].

Despite being polyploids, titan cells can replicate and produce haploid or aneuploid daughter cells with regular size and appearance. The process of “titanization” increases the resistance of cryptococcal cells to oxidative stress and fluconazole, a drug commonly used in the treatment of cryptococcosis [17–19]. The size of fully encapsulated titan cells prevents fungal cells from being phagocytosed by macrophages [21]. Additionally, titan cells induce a Th2 immune response and suppress the autophagy of daughter cells in proximity, which promotes persistence of the infection [22,23]. The ratio of titan cells to normal yeast cells in infected lungs increases with time during chronic and asymptomatic infection [18]. The pheromone-induced process of cryptococcal titanization in MATa and MATα strains is conducted via different G-proteins, which results in increased titan cell production in the presence of pheromones in only the MATa strain [17]. Titan cell formation requires the activation of a cAMP/PKA pathway dependent on adenylyl cyclase *CAC1* and is controlled by a series of positive and negative regulators [17–19,24]. Induction of titan cell formation in vitro can be triggered by exposure of cryptococcal cells to a variety of stimulants including CO_2_, quorum sensing, hypoxia, and exposure to serum [21,25,26].

### Microcells in cryptococcal infections

In addition to regular cells and titan cells, *Cryptococcus* can produce a population of microcells with a thickened cell wall and a cell body diameter not exceeding 2 μm (Fig 1) [27]. These cells were first noted as a distinct population in lungs of infected mice often adjacent to giant (titan) forms [1]. It has been hypothesized that the formation of microcells may be important for the dissemination of fungal cells to the brain and the propagation of immune reconstitution inflammatory syndrome (IRIS) [28]. Analysis of clinical strains of *C*. *neoformans* isolated from patients with HIV indicates a strong correlation of microcell formation with presentation of neurological symptoms, including increased intracranial pressure and vomiting, suggesting an increase in the frequency of successful dissemination to the CNS [27]. The negative correlation of microcell population with acute symptoms suggests a function for this cell population during the later stages of infection [27,29]. Microcells are associated mostly with virulent and hypervirulent strains of *Cryptococcus*. Analysis of several different cryptococcal strains revealed that the gene *SGF29* has a negative impact on the production of the microcells during the infection [30]. Further studies are required to better understand mechanisms that contribute to the formation of microcell phenotype.

### The yeast-to-pseudohypha transformation

Occasionally, *C*. *neoformans* has been observed with a pseudohyphal phenotype (Fig 1). In *Cryptococcus*, pseudohyphae are chains of incompletely separated yeast cells that resemble true hyphae but are separated by constrictions between cells rather than septa [9]. Clinical isolates of *C*. *neoformans* have infrequently been found as pseudohyphae, which can present challenges to diagnosing clinicians [31]. Cryptococcal pseudohyphae are more commonly observed following interactions with environmental predators, such as the protozoan *Acanthamoeba castellanii*, and confer protection against predation by amoebae but are avirulent in mice [32]. It is hypothesized that the transition to a pseudohyphal form is a “biological escape hatch” in the face of danger from environmental threats, such as amoebae or phagocytic immune cells, and that changing the composition of the fungal cell wall, possibly through variation in the amount and type of β-glucan linkages, decreases recognition by predators [32–35]. Various pathways have been identified as important to the yeast–pseudohyphae transition. Lee and colleagues identified limited nitrogen as a trigger of pseudohyphal growth in various *C*. *neoformans* strains through the activity of ammonium permeases *AMT1* and *AMT2* [36]. Crucial to the cryptococcal morphological transition is the RAM pathway, which consists of five proteins and regulates cell polarization [37]. Work by Lin and colleagues showed that pseudohyphal forms of *C*. *neoformans* fared better in their interactions with *Galleria mellonella*, murine macrophages, and environmental predator *A*. *castellanii* than hyphal and yeast forms and that alterations in the RAM pathway and the expression of transcription factor *ZNF2*, which regulates hyphal growth, can affect the response to hosts [35]. Though much has been discovered in recent years, further work on pseudohyphal growth must be done to elucidate the processes behind this cryptococcal morphology.

## Conclusions

Cryptococcal spp. are remarkable in their ability to alter their morphology, being capable of transitioning to giant (titan), micro, hyphal, and pseudohyphal cell types. Most studies of cryptococcal virulence and pathogenesis have focused on the role of yeasts and titan cells in the propagation of infection; few have attempted to elucidate the role of pseudohyphae and microcells, despite their potential clinical relevance. Better understanding of the genetic and molecular mechanisms inducing morphological transitions in cryptococcal cells could enhance our understanding of the role of cellular morphology in pathogenesis and may produce new leads for discovery of novel targets.
